# First case of macrocyclic lactone-resistant *Dirofilaria immitis* in Europe - Cause for concern

**DOI:** 10.1016/j.ijpddr.2024.100549

**Published:** 2024-05-21

**Authors:** Donato Traversa, Anastasia Diakou, Mariasole Colombo, Sohini Kumar, Thavy Long, Serafeim C. Chaintoutis, Luigi Venco, Gianluca Betti Miller, Roger Prichard

**Affiliations:** a Department of Veterinary Medicine, University of Teramo, 64100, Teramo, Italy; b Laboratory of Parasitology and Parasitic Diseases, School of Veterinary Medicine, Faculty of Health Sciences, Aristotle University of Thessaloniki, 54124, Thessaloniki, Greece; c Institute of Parasitology, Faculty of Agricultural and Environmental Sciences, McGill University, Sainte Anne-de-Bellevue, QC, H9X3V9, Canada; d Diagnostic Laboratory, School of Veterinary Medicine, Faculty of Health Sciences, Aristotle University of Thessaloniki, 54627, Thessaloniki, Greece; e Ospedale Veterinario Città di Pavia, 27100, Pavia, Italy; f Ambulatorio Veterinario Farnesina, Via della Farnesina, 25 00135, Rome, Italy

**Keywords:** ddPCR, *Dirofilaria immitis*, Drug resistance, Europe, Heartworm, Macrocyclic lactones, SNP genotyping

## Abstract

Heartworm disease caused by the nematode *Dirofilaria immitis* is one of the most important parasitoses of dogs. The treatment of the infection is long, complicated, risky and expensive. Conversely, prevention is easy, safe, and effective and it is achieved by the administration of macrocyclic lactones (MLs). In recent years, *D. immitis* strains resistant to MLs have been described in Southern USA, raising concerns for possible emergence, or spreading in other areas of the world. The present study describes the first case of ML-resistant *D. immitis* in a dog in Europe. The dog arrived in Rome, Italy, from USA in 2023. Less than 6 months after its arrival in Italy, the dog tested positive for *D. immitis* circulating antigen and microfilariae, despite it having received monthly the ML milbemycin oxime (plus an isoxazoline) after arrival. The microfilariae suppression test suggested a resistant strain. Microfilariae DNA was examined by droplet digital PCR-based duplex assays targeting four marker positions at single nucleotide polymorphisms (SNP1, SNP2, SNP3, SNP7) which differentiate resistant from susceptible isolates. The genetic analysis showed that microfilariae had a ML-resistant genotype at SNP1 and SNP7 positions, compatible with a resistant strain. It is unlikely that the dog acquired the infection after its arrival in Europe, while it is biologically and epidemiologically plausible that the dog was already infected when imported from USA to Europe. The present report highlights the realistic risk of ML-resistant *D. immitis* strains being imported and possibly transmitted in Europe and other areas of the world. Monitoring dogs travelling from one area to another, especially if they originate from regions where ML-resistance is well-documented, is imperative. Scientists, practitioners, and pet owners should be aware of the risk and remain vigilant against ML-resistance, in order to monitor and reduce the spreading of resistant *D. immitis*.

## Highlights

• Macrocyclic lactone-resistant strains of *Dirofilaria immitis* are present in U.S.A.
• This is the first macrocyclic lactones-resistant *Dirofilaria immitis* case in Europe.
• The importance of monitoring the spreading of the phenomenon in Europe is discussed.

## Graphical abstract

**Figure:**
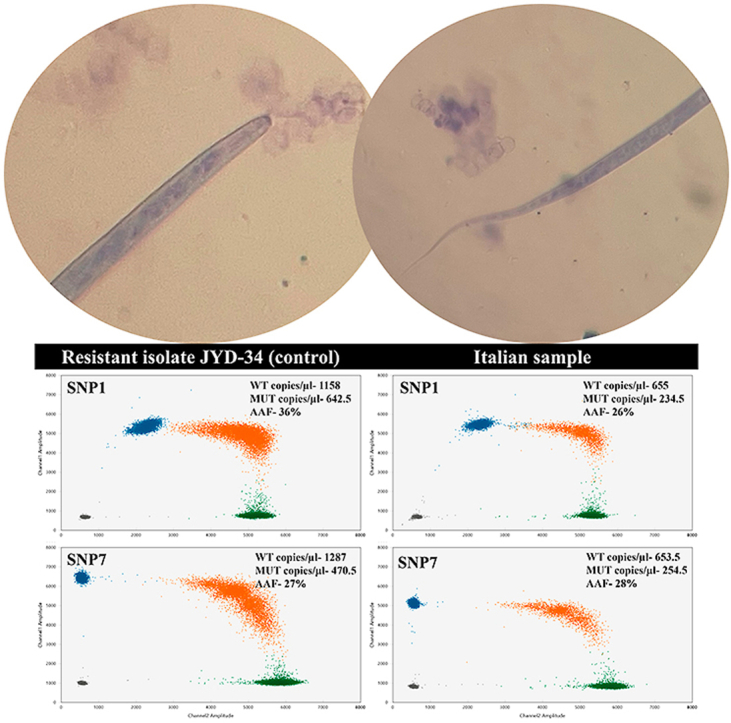

## 1 Introduction

The mosquito-transmitted nematode *Dirofilaria immitis* (Filarioidea: Onchocercidae) is the causative agent of “heartworm disease” (i.e. cardio-pulmonary dirofilariosis) in dogs and other animals. Heartworm disease is one of the most important parasitic diseases in dogs worldwide. It can be potentially lethal for animals, it has zoonotic implications, and its prevalence and geographic distribution are likely spreading around the world due to climate change (Simón et al., 2012; Fuehrer et al., 2021; Geary, 2023).

The main pathological alterations occurring in dogs infected with *D. immitis* are changes of the pulmonary arteries and the lung parenchyma, which cause proliferative arteritis, pulmonary hypertension resulting in alteration of the structure and function of the right ventricle of the heart (*core pulmonale*), and heart failure. Infected dogs may display a range of cardio-pulmonary clinical signs which can lead to the death of untreated animals either due to chronic deterioration of clinical conditions or to the occurrence of acute complications, e.g. the caval syndrome or thromboembolism caused by the death of nematodes (Bowman and Atkins, 2009). The medical treatment of heartworm disease in dogs is a long, expensive, complicated procedure and requires strict compliance of the owners (AHS, 2024; ESDA, 2024). In some cases, surgical removal of the worms is recommended, though this procedure requires skilful operators and appropriate facilities (AHS, 2024).

In areas where dogs act as reservoirs of *D. immitis* and mosquitoes are abundant the parasite poses an important threat also for cats and humans. The infection in cats is characterised by unique traits, e.g. infected cats may suffer from a severe and potentially fatal pulmonary inflammation (heartworm-associated respiratory disease, HARD) or may suddenly die with no clinical signs due to massive thromboembolism or anaphylactic reactions (Lee and Atkins 2010; Bowman and Atkins, 2009; Ryan and Newcomb, 1995). At the same time, the number of human cases is rising and human dirofilariosis is becoming a problem that physicians are increasingly challenged to handle in North America, Europe, Asia, and Australia (Simón et al., 2022). In humans, *D. immitis* usually causes a single granulomatous lesion (“coin lesion”) in the lungs, or it can be found in other sites like the eyes and subcutaneous tissue. These lesions usually alarm physicians and trigger complicated diagnostics, often including surgery, which are stressful for patients and may result in considerable health implications (Simón et al., 2022).

Given the clinical and epizootiological/epidemiological significance of *D. immitis* infection, the prevention of infection in dogs (i.e. the main reservoirs) and cats in both endemic and parasite-free regions is of paramount importance. After the launch in 1987 of the first veterinary product containing the macrocyclic lactone (ML) ivermectin, heartworm disease could be effectively prevented (Hampshire, 2005). To date, all preventive drugs used to control *D. immitis* belong to the ML class, which act efficiently against the third (L3) and fourth (L4) larval stages in a sort of “reach-back” efficacy, i.e. MLs act against parasites that have been already inoculated into the host by the vector in the 30–40 days before dosing (Nolan and Lok, 2012; Diakou and Prichard, 2021).

For marketing authorization all heartworm-preventive formulations must have 100% efficacy demonstrated in experimental trials (Bowman, 2012; Beugnet et al., 2022). Despite this efficacy, some *D. immitis* strains have been proven resistant to MLs, allowing the development of adult parasites in dogs that were under strict preventive schemes (Pulaski et al., 2014; Bourguinat et al., 2015). The phenomenon of *D. immitis* resistance is an alarming condition with the potential to widely jeopardize effective heartworm prevention. Resistance to MLs has emerged in the Lower Mississippi region of the USA and its precise extent cannot be unequivocally defined (Diakou and Prichard, 2021). Recent studies genotyping *D. immitis* isolates from random and suspected cases in Europe have so far failed to identify any resistant genotypes (Diakou et al., 2018; Curry et al., 2022; Kumar et al., 2024). Nevertheless, there is a reasonable concern that resistance to ML may spread to new areas, either by introduction of resistant strains through animal and mosquito movements (e.g., trade of goods) or by an independent evolutionary process, as it occurred in the USA (Diakou and Prichard, 2021).

The present report describes the first documented case of ML-resistant *D. immitis* infection in Europe, with the aim to raise awareness among the research and veterinary communities and to call for the implementation of vigilant monitoring and preventive measures against resistance emergence.

## 2 History, clinical and diagnostic examinations

A ∼2-year-old Australian Shepherd female dog born in New Roads, Louisiana, USA was transferred to its new owners in Italy at the end of May 2023. The dog arrived in Rome on the June 1, 2023, travelling for 3 days through Paris, Venice, and Padua. According to the available information, the dog was neglected by its owner in the USA and then adopted in Italy. No history of veterinary care and heartworm prevention treatment is available for when the dog was living in the USA, except that a serological (antigen) test was performed in April 2023. This test, which detects adult female worms, but not larval stages, was negative. Once arrived in Italy the dog was in a bad general condition and infested by fleas. Thus, the veterinarian who first examined the animal in a veterinary practice in Rome suggested monthly administration of milbemycin oxime + an isoxazoline for flea, tick and endoparasite control.

**Fig. 1:**
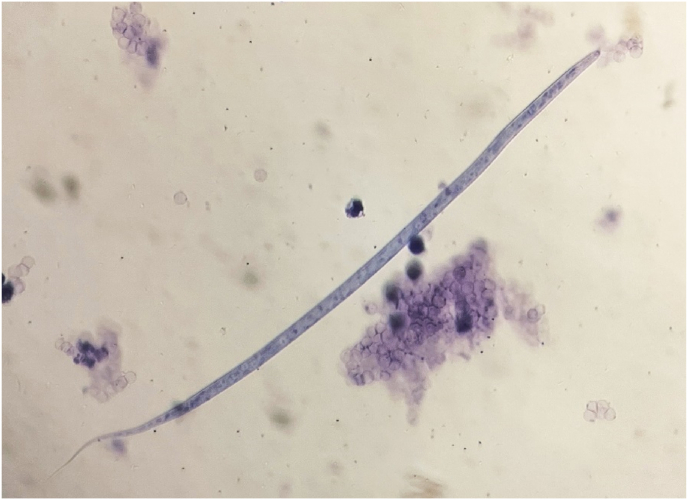
Microfilaria of *Dirofilaria immitis*, displaying characteristic features of the species (pointed cephalic end, straight tail), found at the Knott's test of the dog imported from Louisiana, USA to Italy.

At the end of November 2023, the dog underwent a routine surgery, and among other examinations, a blood sample was sent to a laboratory for diagnostic testing. As the blood was positive in the Knott's test for microfilariae identified as *D. immitis* based on morphometric (length and width) and morphological (anterior and posterior extremities) features (Lindsey, 1965), the dog was referred to a veterinary clinic for a second opinion. The diagnosis of heartworm infection was then confirmed by serological test (IDEXX Snap® 4Dx Plus) and the Knott's method, which scored positive for circulating *D. immitis* antigen and microfilariae (Fig. 1), respectively. Faecal examination by flotation and Baermann methods were negative for parasite elements. The clinical examination was normal, and the owners did not report any clinical signs.

## 3 Investigation of ML-resistance

The fact that the dog had a positive microfilaraemia when the adult heartworm infection was first detected, even though it had been on monthly treatment with a ML for 5 months, could suggest a resistant infection. The fact that the dog had a microfilariae count of 2000 mf/ml when the infection was first detected, despite being on a monthly treatment could be consistent with genotypic and phenotypic ML-resistance of *D. immitis*. It is unlikely that the dog acquired the infection in Italy while it is more likely that it was already infected when it arrived in Italy.

It was impossible to demonstrate persistence of microfilariae despite the monthly administration of a ML from June to October or to further investigate the hypothesis that the dog acquired the infection in Italy. Nevertheless, a suspicion of a ML resistant strain of *D. immitis* was in any case raised, considering the origin of the animal and its preventive treatment history once in Italy.

Immediately after diagnosis, arthropod anti-feeding formulations were applied to the dog and guidelines for home restriction were given to the owners to minimize the possible transmission of the infection, although the diagnosis was made at the end of November, i.e. a month when the transmission risk is low in Italy (Genchi et al., 2011).

The presence of a ML-resistant strain of *D. immitis* was investigated based on a well-established algorithm (Moorhead et al., 2017; Diakou and Prichard, 2021). As the dog was microfilaraemic, the first approach was the microfilariae suppression test (MFST) as previously described (Geary et al., 2011) with recently proposed slight modifications (Diakou and Prichard, 2021). The MFST relied on a first Knott's test with a count of microfilariae per ml (mf/ml) of blood, followed by the administration of topical moxidectin at the dose of 2.5 mg/kg contained in a formulation labelled for microfilaricidal efficacy (Advocate®, Elanco Animal Health). The microfilariae count was repeated 2, 3, and 4 weeks post-dosing to monitor the percentage of microfilariae reduction.

Before the administration of moxidectin, 5 ml of whole blood was collected for molecular investigations. Microfilariae were isolated as previously described (Kumar et al., 2023), harvested by centrifugation (1000×*g*, 5 min) and subjected to DNA extraction with the NucleoSpin XS Tissue kit (Macherey-Nagel, Düren, Germany). The DNA was examined with a droplet digital PCR-based duplex assay targeting four marker positions at single nucleotide polymorphisms (SNP1, SNP2, SNP3, SNP7), as described (Kumar et al., 2023). Briefly, each ddPCR duplex assay reaction mix consisted of 2X ddPCR SuperMix for probes (no dUTP) (Bio-Rad, Hercules, CA, USA), 900 nM each of forward, reverse primers, 250 nM each of FAM and HEX conjugated mutant and wildtype probes, 10 ng *D. immitis* gDNA and synthesized DNA controls at pre-optimized concentrations (Kumar et al., 2023). The reaction mix was combined with droplet generation oil for probes (Bio-Rad) and processed using a QX200™ Droplet Generator (Bio-Rad) resulting in approximately 20,000 nl-sized droplets. These droplets were then transferred to a 96-well plate and sealed to prevent evaporation. PCR was carried out on a C1000 Touch™ thermal cycler (Bio-Rad) following the manufacturer's instructions. A droplet cut-off greater than 12000 was applied during data analysis (BioRad, 2018). The ddPCR data was subsequently analysed using QuantaSoft™ Analysis Pro software (Bio-Rad, version 1.0.596). The number of droplets showing wildtype or mutant type (either wildtype or mutant only) or wildtype plus mutant type was calculated based on Poisson distribution using this software. The percentage of alternate allele frequency (AAF) was determined by calculating the ratio of mutant target copies to total DNA copies (mutant + wildtype). Assays were performed in duplicate.

## 4 Results

**Fig. 2:**
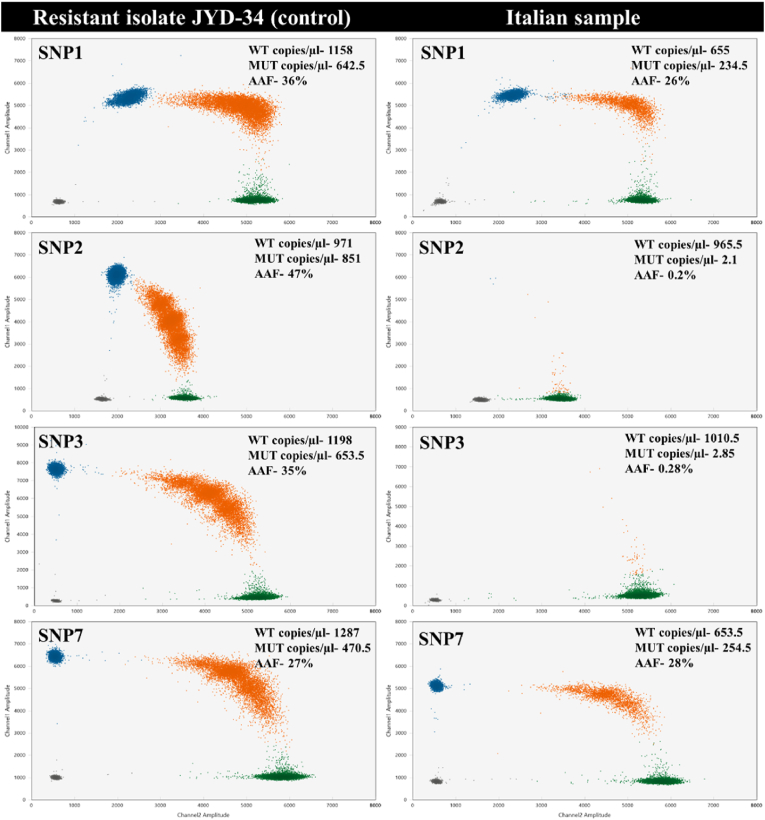
Two-dimensional (2D) droplet-digital polymerase chain reaction (PCR) plots illustrate the amplification of wildtype and alternate nucleotide (mutant) targets at respective single nucleotide polymorphism (SNP) positions in *D. immitis*. The absolute quantification of wildtype and mutant targets across SNP1, SNP2, SNP3, and SNP7 positions is depicted in a well characterized ML resistant isolate of *D. immitis,* JYD-34 (A), and Italian canine sample (B). For each plot, the amplitude in Channel 1 (y-axis) and Channel 2 (x-axis) represents the relative fluorescence of each droplet detected at the FAM and HEX channel, respectively. Green and blue droplets indicate positive droplets for wildtype and alternate allele targets, respectively, orange droplets indicate droplets having both wildtype and mutant targets and black droplets indicate negative droplets with no target amplification. Targets detected are indicated in number of copies per μl of the PCR reaction volume. This was used to calculate the percentage of alternate allele frequency (AAF). (For interpretation of the references to colour in this figure legend, the reader is referred to the Web version of this article.)

At baseline (i.e. pre-treatment with moxidectin) microfilariae count was 2000 mf/ml of whole blood. At the MFST the counts were 936 mf/ml, 2920 mf/ml, and 2000 mf/ml on 2-, 3-, and 4 weeks post moxidectin administration, respectively. The genetic analysis showed that microfilariae had a ML-resistant genotype at SNP1 and SNP7 positions, while at SNP2 and SNP3 wildtype SNP frequencies predominated (Fig. 2).

## 5 Discussion

The identification of specific SNPs associated with susceptible and resistant phenotypes of *D. immitis* (Bourguinat et al., 2017; Ballesteros et al., 2018) is a successful approach which has recently led to the development of the ddPCR duplex assay herein applied (Kumar et al., 2023). This assay is a powerful and reliable tool for assessing the genetic evidence of resistance of individual isolates on a clinical case level (Kumar et al., 2023). A discordance between SNP1 and SNP2 in analysed samples, as also detected in the present case, is occasionally observed. In a recent study, 2 adult worms of the USA-resistant laboratory-maintained isolate “WildCat” displayed a resistant genotype at the SNP2 position but a susceptible genotype at SNP1 (Kumar et al., 2024). A discrepancy, e.g., between SNP1 and SNP2 has also been observed on rare occasions in other microfilarial populations analysed using the MiSeq sequencing platform (Prichard, unpublished data). In the case presented herein the overall SNP analysis indicates a ML-resistant infection. This has been also confirmed by the results of the MFST, where microfilariae counts were reduced by only 53% on week 2, rose over the initial counts by 46% on week 3, and returned to the initial counts (0% overall reduction) by week 4. In the MFST a susceptible strain is expected to display microfilariae counts reduction of ≥75% (Diakou and Prichard, 2021). Therefore, the dog was infected by an unequivocally ML-resistant strain of *D. immitis*.

The first reports of resistance to MLs in *D. immitis* -at that time referred to as loss of efficacy (LOE) cases-were described in 1998, only 10 years after MLs were first introduced to the market as heartworm preventives (Hampshire, 2005). Although most of those reports were attributed to a lack of owner compliance (Atkins et al., 2014), a few years later *D. immitis* strains from the Lower Mississippi region that displayed a resistant phenotype, were confirmed as truly resistant to ML in all possible ways, i.e. genetically, *in vitro*, and clinically (Bourguinat et al., 2011, 2015; Pulaski et al., 2014). Since then, ML-resistance has been repeatedly recorded and confirmed in the wider area of the Lower Mississippi River Valley region. Inevitably, it has been recurrently argued that further spread of ML-resistant heartworms is likely, if not fated (Diakou and Prichard, 2021; Prichard 2021; Savadelis et al., 2022).

It should be kept in mind that there is a high chance that *D. immitis* strains resistant to ML may have always naturally existed but were very rare. It is plausible that the idea of MLs being 100% effective in preventing heartworm is a misconception caused by the ML registration studies that were conducted using only a few, experimentally maintained, *D. immitis* isolates. It is reasonable that a high level of efficacy is a prerequisite when heartworm prevention is in question, justifying the high standards of registration studies (Beugnet et al., 2022). Nevertheless, the factual efficacy of any molecule would be accurately assessed only if thousands of different “wild” strains of the parasite were tested, which, of course, is practically unfeasible (Prichard, 2021).

To date, several factors may have supported, and may influence in the future, the emergence and spreading of ML-resistance in *D. immitis*. Key modulators are selection pressure, inbreeding phenomena, refugia (i.e. the portion of parasites that are not in contact with a drug), infection pressure, the effect of MLs on different *D. immitis* stages, and transportation of resistant strains in new areas (Diakou and Prichard, 2021; Prichard, 2021). Selection pressure may occur every time sub-preventive levels of a ML are present in the blood of a dog. According to pharmacokinetic studies, this happens a few days after dosing, as the concentration of ML decreases rapidly (Prichard, 2021). Resistance to MLs is a polygenic trait and it is plausible that resistance intensity varies in different phenotypes (Diakou and Prichard, 2021). Thus, when sub-preventive concentrations act on developing L3/L4 of a less susceptible genotype they may survive and even develop to adulthood if the next ML dose is missed or delayed. In practical terms, missed or delayed doses are extremely likely in thousands of dogs under monthly chemoprevention in enzootic areas. Such an accumulated, monthly selection pressure may have resulted in resistant emergence after only 10 years of ML use as heartworm preventives. The lifecycle of *D. immitis*, relying on transmission *via* mosquitos, intrinsically favours the emergence and spread of ML resistance. Vector transmission enhances inbreeding phenomena that result in the predominance of some genotypes. In fact, a mosquito is likely to acquire and later transmit genetically identical or very similar parasites produced by a relatively small number of adult nematodes in a host. In case these larvae are tolerant to some extent to MLs, they may develop into adults even if prevention is applied to the next host. This scenario, if repeated, may progressively foster parasite generations with resistance to MLs (Churcher et al., 2008; Prichard, 2021). The lack of free developmental stages in *D. immitis* limits refugia mainly to parasites in vertebrates that are not under prevention, while the proportion of the population in mosquitoes is minimal. As a result, refugia are suppressed and the selection for resistance is enhanced where prevention in domestic dogs is extensively applied (Wolstenholme et al., 2015; Diakou and Prichard, 2021). In these areas, infection pressure associated to high-density and stable populations of mosquitoes is an additional drive towards resistance establishment, because resistant genotypes may be intensively transmitted (Diakou et al., 2014; Prichard, 2021). Finally, MLs have an effect also on microfilariae and adult filariae and may kill them or impair their reproduction when administered for long periods of time to infected dogs (McCall, 2005; McTier et al., 2017; Prichard, 2021). This may further favour the domination of resistant strains in a *D. immitis* population.

Resistant strains may be transferred to other locations *via* mosquito and/or dog importation and travel. In fact, the first ultimately confirmed case of ML-resistance in *D. immitis* was from a dog which had been imported into Canada from Louisiana, USA (Bourguinat et al., 2011). Indeed, the potential of mosquitoes as spreaders of resistant strains is limited because: i) these insects do not travel actively far away from their environment (Prichard, 2021), and ii) their passive transfer by transportation means could play a role only if a considerable number of infected female mosquitoes would be successfully transferred. Conversely, resistance spreading by infected dogs is a realistic threat, because any microfilariae act as a constant source of infection for local mosquitoes. This could be the scenario of the case described here, as the most realistic hypothesis is that the dog was already infected when imported into Italy from the USA. Although it is not known to what extent, if any, the dog had the chance to infect local mosquitoes, this case is exactly the scenario described as “risk of resistance importation” in the recent literature (Wolstenholme et al., 2015; Diakou and Prichard, 2021; Prichard, 2021; Savadelis et al., 2022).

Knowledge of the history of this dog is fragmentary. The dog was born and lived for around 2 years in Louisiana, an area of high occurrence of resistant *D. immitis* strains (Prichard, 2021). It was negative for heartworm antigen in April 2023, but it was not tested again before its travel to Europe. The antigen test only reveals the presence of adult female worms, thus immature stages (i.e. migrating L4, pre-adults L5) are not detected with this test. It is not known if the dog was receiving any heartworm prevention while residing in Louisiana. It was a neglected animal according to the descriptions of its new owners, so it is likely that it received inconsistent or no heartworm prevention in the USA. Microfilariae were detected at the end of November, after being almost 6 months in Italy. It is impossible to unequivocally determine if the dog was infected in the USA or acquired the infection immediately after it arrived in Europe. However, it is plausible that the dog acquired the infected in Louisiana for three reasons. First, the prepatent period for *D. immitis* is 6–9 months (Bowman and Atkins 2009) and microfilariae were first detected a few days before 6 months had passed since it arrived in Italy. Second, dogs living in central and southern Italy are often not under preventives, i.e. a condition which does not spur selection pressure and resistance development in a given area. Finally, to date, there is no record of any autochthonous cases of ML-resistant *D. immitis* in Italy or Europe in general.

At the same time, it is impossible to know when the dog turned microfilaraemic. It is worth noting that during its travel it spent a few hours in Atlanta, Paris, and Venezia, and 3 days in Padua before arriving in Rome. While there is a possibility that mosquitoes in these transit locations have acquired microfilariae of a resistant genotype, the likelihood of the resistant strain becoming established in these areas is low. In fact, the resistant genome would be likely diluted within the pool of susceptible strains in the receiving area, particularly if there is a significant number of hosts that are not under prevention (i.e., allowing large refugia) (Diakou and Prichard, 2021). However, the selection pressure may trigger resistance establishment when a microfilaraemic dog is a continuous source of resistant parasites where preventives are administered (Diakou and Prichard, 2021). This could be the case of Italy, where i) northern regions are historically hyper-enzootic for *D. immitis* with a high density of mosquitoes and extensive use of MLs, and ii) an expansion of *D. immitis* towards central and southern areas justifying a wider use of preventives has been recently shown (Mendoza-Roldan et al., 2020). Therefore, the establishment of ML resistance in Italy, especially if a critical number of mosquitoes become infected with the resistant strain, could be considered a realistic threat.

It is advantageous that the drug used for treating the infection, i.e. melarsomine dihydrochloride, is not a ML. Therefore, it safeguards the elimination of adult parasites despite any ML-resistant genotype. The so called “slow kill” treatment protocol, i.e., the administration of repeated MLs at chemopreventative dosages that gradually reduce adult heartworms’ viability, would not be effective against resistant strains. Moreovoer, this approach would permit the resistant strain to be transmitted and likely enhance the resistant character due to continuing selection pressure (Diakou and Prichard, 2021). Furthermore, it has been suggested that “slow kill” can promote ML resistance development in new areas, as the administered ML doses kill only susceptible parasites, leaving unaffected any resistant worms present in the population (Geary et al., 2011). Accordingly, although the “slow kill” protocol could be an alternative where melarsomine is unavailable or unaffordable, adulticides should always be the preferred choice.

In the case described here, after the results of the MFST, the dog was treated according to the guidelines of the American Heartworm Society (AHS, 2024) and the European Society of Dirofilariosis and Angiostrongylosis (ESDA, 2024). The administration of doxycycline eliminates the filarial endosymbiont *Wolbachia pipientis*, thereby reducing its pathological effects following the death of the filariae, but what is relevant in the case of a resistant strain, the elimination of this endosymbiont in microfilariae impairs their development in a new host, ensuring the discontinuation of resistance spreading (McCall et al., 2014). For the same reason, protection against mosquito bites is also important in the management of a suspected or confirmed resistant infection.

In conclusion, the prevention of *D. immitis* infection is crucial, because of its considerable impact on animal health and welfare and zoonotic implications. MLs are the only molecules currently available and licenced for heartworm disease prevention. Although ML-resistant strains of *D. immitis* have been, until now, largely restricted to the Lower Mississippi region of the USA, the risk of their expansion in new areas of the world is realistic, as indicated by the present report. Given that it is highly plausible that the dog arrived in Italy already infected with the resistant *D. immitis* strain, the here presented first case ever of ML-resistant *D. immitis* infection in Europe is a demonstration of the factual risk of importing drug resistant heartworm genotypes into Europe. Therefore, dogs originating from areas where ML-resistance is well-documented and established should be closely monitored by appropriate examinations before their travel and for 6–9 months after their arrival to the new location. Diagnostics should always include the Knott's test, which has the potential to reveal the infection even when a few resistant adult worms have developed in the host. In fact, microfilariae may be produced by a single couple of resistant adults even when serological tests are negative due to a low number of adult females (Diakou and Prichard, 2021).

Monitoring the occurrence of ML-resistant strains of *D. immitis* is critical for the surveillance of this dynamic phenomenon and the control of its expansion. Accordingly, scientists, practitioners and pet owners should remain constantly vigilant and work synergistically.

## CRediT authorship contribution statement

**Donato Traversa:** Writing – review & editing, Writing – original draft, Validation, Formal analysis, Data curation, Conceptualization. **Anastasia Diakou:** Writing – review & editing, Writing – original draft, Investigation, Data curation. **Mariasole Colombo:** Writing – original draft, Methodology, Investigation, Data curation. **Sohini Kumar:** Methodology, Investigation. **Thavy Long:** Methodology, Investigation. **Serafeim C. Chaintoutis:** Methodology, Investigation. **Luigi Venco:** Validation, Formal analysis, Data curation. **Gianluca Betti Miller:** Methodology, Investigation. **Roger Prichard:** Writing – review & editing, Writing – original draft, Validation, Supervision, Formal analysis, Data curation.

## Declaration of competing interest

The authors declare no conflict of interest.
